# Efficacy of Immediate Switching from Bicalutamide to Flutamide as Second-Line Combined Androgen Blockade

**DOI:** 10.1155/2016/4083183

**Published:** 2016-07-14

**Authors:** Yumiko Yokomizo, Takashi Kawahara, Yasuhide Miyoshi, Masako Otani, Shoji Yamanaka, Jun-ichi Teranishi, Kazumi Noguchi, Masahiro Yao, Hiroji Uemura

**Affiliations:** ^1^Department of Urology, Yokohama City University Graduate School of Medicine, Yokohama 2360004, Japan; ^2^Department of Urology, Yokohama City University Medical Center, Yokohama 2320024, Japan; ^3^Department of Diagnostic Pathology, Yokohama City University Medical Center, Yokohama 2320024, Japan; ^4^Department of Pathology, Yokohama City University Hospital, Yokohama 2360004, Japan

## Abstract

We determined whether prostate specific antigen (PSA) would decrease with immediate antiandrogen switching from bicalutamide (BCL) to flutamide (FLT) in patients receiving combined androgen blockade for advanced prostate cancer. From 2002 to 2006, 20 patients who showed PSA failure after first-line hormonal therapy with a luteinizing hormone-release hormone (LH-RH) agonist and BCL were enrolled. All patients were immediately switched from BCL to FLT, administered with an LH-RH agonist, as second-line combined androgen blockade (CAB). We evaluated the PSA response to second-line CAB. Eight patients (40%) were responsive, showing PSA decreases of at least 50%. The median (range) duration of the PSA response was 18.4 (3–26) months. Second-line CAB using FLT was effective in 40% of patients who received first-line CAB using BCL. The lower Gleason scores at the initial prostate biopsy probably reflect the response to second-line CAB. Responders showed significantly better OS and CSS in the determination of any PSA decline and 40% PSA decline. The median OS duration in nonresponders and responders (40% PSA decline) was 1433 days versus 3617 days. It is concluded that an immediate switch from BCL to FLT is effective for some CRPC patients after first-line CAB using BCL.

## 1. Introduction

The introduction of prostate specific antigen (PSA) as a clinical marker for prostate cancer in the late 1980s has increased the number of men who are diagnosed with the disease. Recently, prostate cancer diagnoses have been increasing at a faster rate in Japan. Since Miyamoto et al. first reported the use of hormonal therapy (androgen deprivation therapy) in the treatment of prostate cancer in 1941, the concept of androgen ablation or the blockade of androgenic actions has remained a critical and universal treatment approach, especially for patients with advanced prostate cancer [1]. The current hormonal therapy includes surgical or medical castration, including luteinizing hormone-release hormone (LH-RH) analogues, with or without antiandrogen agents. Combined androgen blockade (CAB) using LH-RH analogues and antiandrogen agents has been regarded as an ideal hormonal therapy. Despite the cost and toxicity of antiandrogen agents, CAB therapy has been established as the first-line hormonal therapy in Japan because Japanese health insurance covers the costs. Furthermore, the side effects of antiandrogen agents, such as hepatotoxicity, are widely recognized and thus detected early and carefully treated.

Two oral nonsteroidal antiandrogens, flutamide (FLT) and bicalutamide (BCL), are currently available in Japan. With respect to the adverse effects of nonsteroidal antiandrogens, diarrhea and hepatotoxicity have been reported to occur more frequently with FLT than with BCL [2, 3]. Thus, BCL has been more widely used than FLT as a first-line CAB agent in Japan. Although hormonal therapy in patients with metastatic prostate cancer generally provides good initial efficacy, with response rates as high as 80–90%, most patients who receive hormonal therapy develop resistance within several years. It has recently been reported that chemotherapy using docetaxel improved the survival of patients with castration-resistant prostate cancer (CRPC) [4]. However, the median survival after the development of CRPC is reported to be 16–18 months, even when docetaxel-based chemotherapy is administered [5]. Accordingly, it is important that the effective duration of hormonal therapy can be prolonged by employing various forms of hormonal manipulation, possibly improving the prognosis of patients with advanced-stage prostate cancer.

Considering that there are some differences in the molecular actions of BCL and FLT, switching from one of these antiandrogens to the other as a second-line CAB agent is thought to be necessary when first-line CAB therapy fails. In the present study, we analyzed the clinical data of patients who were treated with BCL as a first-line CAB agent and who subsequently switched to FLT as a second-line CAB agent.

## 2. Patients and Methods

### 2.1. The Patients and the Regimen for Switching Antiandrogen Agents

A total of 20 patients were enrolled in this study. All of the patients had been pathologically diagnosed with prostate cancer at Yokohama City University Hospital or Yokohama City University Medical Center during the 2002–2006 period. The pathological findings were determined by a prostatic needle biopsy examination, and clinical staging was determined according to the tumor node metastasis (TNM) classification using computed tomography, magnetic resonance imaging, and bone scintigraphy. The Gleason score was calculated according to the ISUP 2005 classification. We retrospectively analyzed the data.

All of the patients received an LH-RH analogue (goserelin acetate or leuprorelin acetate), which was injected subcutaneously every 4 or 12 weeks, plus an antiandrogen (80 mg/day BCL) as a first-line CAB therapy. After the confirmation of biochemical (PSA) failure under first-line CAB treatment, BCL was discontinued and the patient was immediately switched to FLT (250 mg/day) as a second-line CAB agent. The LH-RH analogue injections were continued after biochemical failure. The PSA levels were measured approximately once every 4 weeks using a chemiluminescent enzyme immunoassay (CLEIA) by both hospital laboratories. After the confirmation of second-line CAB failure, the administration of FLT was discontinued for at least 8 weeks to investigate the existence of antiandrogen withdrawal syndrome (AWS).

### 2.2. Evaluation of the Response to Treatment

In patients undergoing second-line CAB therapy, a response was defined as a 40% or 50% decrease in the PSA level after the start of the second-line CAB therapy. The response duration was defined as the time from the start of the second-line CAB therapy until PSA failure, which was defined as three successive PSA elevations. The existence of AWS was defined as a ≥50% decrease in the PSA level after discontinuing FLT.

The PSA responses after first-line CAB therapy were as follows: (1) a response was defined as the normalization of the PSA level (<4.0 ng/mL); (2) a partial response (PR) was defined as a <50% decrease in the PSA level in comparison to the initial PSA level (with the PSA level remaining at >4.0 ng/mL); (3) progressive disease (PD) was defined as a >25% increase in the PSA level in comparison to the initial level; (4) no change (NC) was defined as a PSA level that remained between a PR and PD.

### 2.3. Statistical Analyses

Statistical analyses were performed using the Mann-Whitney *U* test and Fisher's exact probability test. *P* values of <0.05 were considered to indicate statistical significance in all tests.

## 3. Results

The characteristics of the 20 enrolled patients are shown in Table 1. The mean pretreatment PSA level was 760 ng/mL (range 17.2–5740 ng/mL). The patients' clinical stages were classified as T2 (*n* = 1), T3 (*n* = 11), and T4 (*n* = 8). The patients' Gleason scores were 6 (*n* = 1), 7 (*n* = 5), 8 (*n* = 4), 9 (*n* = 7), 10 (*n* = 1), and unknown (*n* = 2). Seventeen of the 20 enrolled patients (85%) showed a CR based on their PSA levels after first-line CAB therapy; the remaining 3 patients showed a partial response (PR). Only one patient (5%) developed liver dysfunction (grade 2), despite showing a response to the second-line CAB therapy. The mean duration of the first-line CAB therapy (with BCL) was 20.1 months (median, 14.5; range, 7–50 months).

The eight (40%) patients who showed a >50% decrease in their PSA levels in response to second-line CAB therapy using FLT were considered to be responders. The mean duration of the PSA responses was 18.4 months (median, 21.5; range, 3–26 months). At the time of writing, 2 of these 8 responders remain responsive. Two (10%) of the 20 enrolled patients showed a brief moderate PSA response (47.2% and 45.5%) after second-line CAB therapy, indicating that the overall response rate of the patients who received second-line CAB therapy was 50% (Table 2). Only one patient among the responders died from prostate cancer.

Several factors were compared to determine the differences between responders and nonresponders. As shown in Table 2, there was a significant difference (*P* = 0.041) in the Gleason scores of the two groups, with the responders having a significantly lower Gleason score than the nonresponders. In terms of pathological differentiation, however, the responders included a greater number of patients with moderately differentiated adenocarcinoma (*n* = 5) and fewer patients with poorly differentiated adenocarcinoma (*n* = 1) than the nonresponders. In contrast, 3 nonresponders had moderately differentiated adenocarcinoma and 7 had poorly differentiated adenocarcinoma (*P* = 0.041) (data not shown). The serum PSA level at the start of first-line CAB therapy, the nadir PSA level after first-line CAB therapy, and the duration from the start of first-line CAB therapy to the nadir PSA level were not significantly associated with the PSA response to second-line CAB therapy. Furthermore, the mean PSA level at the time of the diagnosis and the mean duration from first-line CAB therapy to the nadir PSA level after first-line CAB therapy did not differ significantly between the responders and the nonresponders. However, the PSA levels at the start of second-line CAB therapy (*P* = 0.09) tended to be lower among the responders than the nonresponders (Table 2). When a response was defined as a >40% decrease in the PSA level in comparison to the start of second-line CAB therapy, the PSA levels (mean 4.02 ng/mL) of the responders at the start of second-line CAB therapy were significantly lower than those of the nonresponders (mean, 29.7 ng/mL; *P* = 0.023) (Table 3). On the other hand, there was no significant difference in the clinical stages of the patients in the groups (data not shown). AWS occurred in no responders and 1 nonresponder (8.3%) after the start of second-line CAB therapy.

**Figure 1:**
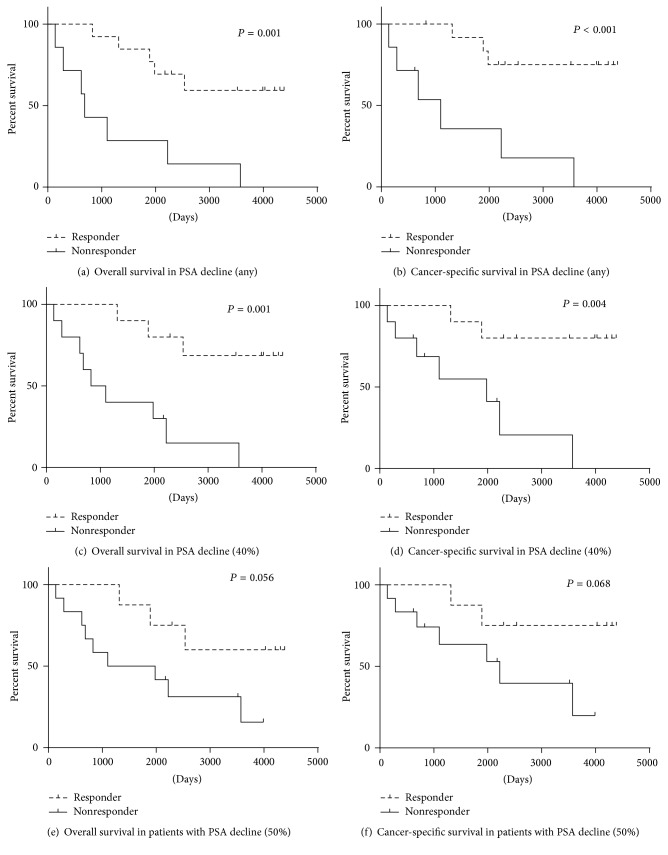
Overall and cancer-specific survival in the responders and nonresponders.

In this study cohort, the responders showed better overall and cancer-specific survival than the nonresponders (Figure 1). The median OS in nonresponders and responders was as follows: 1,232 days versus 3,315 days among patients with any PSA decline; 1,433 days versus 3,617 days among patients whose PSA levels declined by 40%; and 1,883 days versus 3,412 days among patients whose PSA levels declined by 50%. The median CSS in the nonresponders and responders was as follows: 1,414 days versus 3,721 days among patients with any PSA decline; 1,736 days and 3,828 days among patients whose PSA levels declined by 40%; and 2219 days versus 3689 days among patients whose PSA levels declined by 50%. The responders with any PSA decline and those with a 40% PSA showed a significantly better OS and CSS. Although the difference did not reach statistical significance, the responders with a 50% PSA decline tended to show a better prognosis.

## 4. Discussion

In Japan, CAB therapy has come to be widely used as a first-line hormonal treatment for patients with prostate cancer, especially those with advanced-stage disease. CAB includes LH-RH analogues and antiandrogen agents, including steroidal and nonsteroidal antiandrogen agents. In the present study, we treated prostate cancer patients with an LH-RH analogue and BCL as a first-line CAB therapy and investigated the response rate, as reflected by a decrease in the PSA level, when FLT was administered instead of BCL as a second-line CAB therapy. An important feature of this study was that BCL was switched to FLT immediately after three successive PSA elevations were observed with the first-line treatment because BCL was found to have a longer half-life than FLU. The response rate (defined by a marked decrease in the PSA level) of 40% was similar to previous reports [6–8].

Miyake et al. reported that however the response rate in patients receiving FLT as a second-line CAB agent was 22% and that AWS was recognized in 13% of patients who received BCL as a first-line CAB agent [9]. In the present study, changes in the subjects' PSA levels were monitored and FLT was not administered for 8 weeks after PSA failure. Overall, 13% of our patients showed a PSA decrease of >50%. The only patient with AWS in our present series was a nonresponder after second-line CAB. Because BCL was immediately switched to FLT when PSA failure was recognized during first-line CAB therapy, the decrease in the PSA level that was observed at that time might have been due to AWS, which was caused by the discontinuation of BCL. However, Schellhammer et al. reported that the PSA nadir levels after AWS were higher than those that were observed during the initial CAB treatment [10]. In the present study, the PSA nadir levels that were observed with second-line CAB therapy in the responder group (*n* = 7) were lower (median, 0.014; range, 0.003–7.31 ng/mL) than those that were observed with the first-line CAB therapy (median, 0.051; range, 0.012–14.7 ng/mL); thus, the PSA decrease that occurred with second-line CAB was most likely attributable to the administration of FLT.

It is important to identify the factors that predict a response to second-line CAB therapy. Previous reports have indicated that the presence of bone metastasis at the time of the diagnosis, the duration of the response to the initial hormonal therapy, and the time to progression after first-line CAB therapy are predictors of a response to a second-line CAB therapy [7, 9]. Our data do not support the use of these factors as predictors of responsiveness. In contrast, Kojima et al. found that responders tended to have lower PSA levels at the start of second-line CAB therapy [6], which is in agreement with our findings. The current results indicate that a good PSA response to second-line CAB therapy was observed at a significantly higher rate among the patients who had lower Gleason scores at the time of the initial prostate biopsy. Importantly, the present study identified a pathological factor that was a response predictor.

A greater understanding of the molecular mechanism underlying the response to the change in antiandrogen therapy is expected to lead to the elucidation of the molecular changes that take place when hormone sensitive patients develop CRPC. It has been suggested that androgen receptors (ARs) and neuroendocrine cells may play pivotal roles in the development of CRPC [11, 12]. Although it is still unclear how prostate cancer growth changes from being hormone dependent to hormone independent, AR activation via amplification and mutation and/or related AR signaling is considered to play a key role [1, 13, 14]. Among the other factors that are related to the activation of the ARs, cofactors are considered to be important for AR transactivation. Some AR cofactors are known to be upregulated or downregulated in patients with prostate cancer [15]. The deregulation of the expression of some AR cofactors, as well as interactions between AR and these cofactors, has been demonstrated in numerous studies [16]. For example, the expression of ARA55 in CRPC patients is lower than that in patients with benign prostatic hyperplasia (BPH) or untreated prostate cancer. Moreover, higher ARA55 expression levels are associated with shorter recurrence-free survival and overall survival in CRPC patients [17].

In the present study, there was no significant difference in the duration for which BCL was effective between responders and nonresponders to FLT as a second-line CAB agent. The mean period of effective BCL treatment was 23 months (median, 18 months; range, 11–38 months), while the mean period of effective FLT treatment was 18.4 months (median, 14 months; range, 7–50 months). These results may be attributable to differences in individual genes, such as AR abnormalities in prostate cancer cells. More importantly, AR mutations may be implicated in the effectiveness of second-line CAB therapy. A recent report revealed that an AR (H874Y) mutation significantly enhanced binding and transactivation activity with p160 (a coactivator protein) [18]. BCL acts as an antagonist in prostate cancer cells with an AR (T877A) mutation but FLT does not [19]. In contrast, FLT behaves as an antagonist in patients with two AR mutations (W741C and W741L) [20]. Furthermore, combination treatment of FLT with an LH-RH analogue was demonstrated to exert an inhibitory effect on the secretion of adrenal androgens, including androstenedione and dehydroepiandrosterone [21]. Furutani et al. reported that FLT also abrogated the androgen-induced stabilization of AR with the inhibition of the transactivation function of AR more strongly than BCL [22]. Thus, the accumulation of data will likely support the hypothesis that FLT is effective as a second-line CAB agent in CRPC patients who previously received BCL.

Although nilutamide, which is a nonsteroidal antiandrogen with a chemical structure that is distinct from BCL and FLT [23], is not available in Japan, its efficacy as a secondary hormonal therapy after treatment with BCL or FLT has been reported [24–26]. The studies of nilutamide obtained response rates in CRPC patients that ranged from 40 to 64%. Because the datasets of such studies (including our own study) have been very small, the possibility of biases, including a potential bias in patient selection, cannot be denied. Another limitation of these studies is that they may include incomplete datasets. A randomized study is necessary to evaluate the survival benefit obtained when different antiandrogens are used to treat patients with CRPC.

## 5. Conclusion

Second-line CAB therapy with FLT was effective in 40% of patients who received first-line CAB therapy with BCL. The mean duration of the response to second-line CAB therapy was 24.1 months, and the Gleason score at the initial prostate biopsy probably reflected the response to second-line CAB therapy. A further detailed study with a long follow-up period in which a large cohort is treated using carefully ordered hormonal therapies is needed.

## Figures and Tables

**Table 1:** Patient characteristics (*n* = 20).

Mean age, years (range)	70.9	(52–92)
Mean PSA at the diagnosis, ng/mL (range)	760	(17.2–5740)
Clinical stage, *n* (%)		
T2	1 (5)	
T3	11 (55)	
T4	8 (40)	
Gleason score, *n* (%)		
≤6	1 (5)	
7	3 (15)	
8	4 (20)	
9	11 (55)	
10	1 (5)	
Response to first-line CAB therapy, *n* (%)		
Response	17 (85)	
PR	3 (15)	

CAB: combined androgen blockade; PR: partial remission.

**Table 2:** The clinical factors in the responders and nonresponders to second-line CAB therapy.

	Responders (50% PSA change)	Nonresponders	*P* value
Number of patients (%)	8 (40%)	12 (60%)	—
Clinical stage, *n* (%)			
T2	1 (12.5)	0 (0)	
T3	5 (62.5)	6 (50)	
T4	2 (25.0)	6 (50)	0.25
Gleason score, *n* (%)			
6	1 (12.5)	0 (0.0)	
7	1 (12.5)	2 (16.7)	
8	2 (25.0)	2 (16.7)	
9	4 (50.0)	7 (58.4)	
10	0 (0)	1 (8.3)	0.654
Mean PSA at the diagnosis, ng/mL (range)	487.5 (23.8–2350)	941 (17.2–5740)	0.94
Mean nadir PSA after first-line CAB therapy, ng/mL (range)	2.06 (0.003–14.7)	2.67 (0.011–16.8)	0.11
Mean duration to nadir PSA after first-line CAB, months	12.3 (3–26)	13.8 (2–48)	0.64
Mean duration of first-line CAB therapy, months	23 (11–38)	18.4 (7–50)	0.32
Mean PSA at the start of second-line CAB therapy, ng/mL (range)	4.88 (0.116–20.7)	24.8 (0.114–116.3)	0.09
Mean duration of the response to second-line CAB therapy, months	18.4 (3–26)	—	—
AWS	0	1	—

CAB: combined androgen blockade; AWS: antiandrogen withdrawal syndrome.

**Table 3:** The clinical factors in the responders (>40% PSA decrease) and nonresponders to second-line CAB therapy.

	Responders (40% PSA change)	Nonresponders	*P* value
Number of patients (%)	10 (50%)	10 (50%)	—
Mean PSA at the diagnosis, ng/mL (range)	971.1 (23.8–5740)	548.9 (17.2–2590)	0.6
Mean nadir PSA after first-line CAB therapy, ng/mL (range)	1.69 (0.003–14.7)	3.16 (0.011–16.8)	0.059
Mean duration to nadir PSA after first-line CAB, months	13.9 (3–31)	12.4 (2–48)	0.32
Mean PSA at the start of second-line CAB therapy, ng/mL (range)	4.02 (0.116–20.7)	29.7 (0.114–116.3)	0.023
Mean duration of the response to second-line CAB therapy, months	17.2 (3–26)	—	—

CAB: combined androgen blockade.
